# H4K12ac is regulated by estrogen receptor-alpha and is associated with BRD4 function and inducible transcription

**DOI:** 10.18632/oncotarget.3439

**Published:** 2015-01-31

**Authors:** Sankari Nagarajan, Eva Benito, Andre Fischer, Steven A. Johnsen

**Affiliations:** ^1^ Department of General, Visceral and Pediatric Surgery, University Medical Center Göttingen, Göttingen, Germany; ^2^ Department of Psychiatry and Psychotherapy, University Medical Center Göttingen, Göttingen, Germany; ^3^ Research Group for Epigenetics in Neurodegenerative Diseases, German Center for Neurodegenerative Diseases (DZNE) Göttingen, Göttingen, Germany

**Keywords:** Histone acetylation, bromodomain, estrogen, epigenetics, chromatin

## Abstract

Hormone-dependent gene expression requires dynamic and coordinated epigenetic changes. Estrogen receptor-positive (ER^+^) breast cancer is particularly dependent upon extensive chromatin remodeling and changes in histone modifications for the induction of hormone-responsive gene expression. Our previous studies established an important role of bromodomain-containing protein-4 (BRD4) in promoting estrogen-regulated transcription and proliferation of ER^+^ breast cancer cells. Here, we investigated the association between genome-wide occupancy of histone H4 acetylation at lysine 12 (H4K12ac) and BRD4 in the context of estrogen-induced transcription. Similar to BRD4, we observed that H4K12ac occupancy increases near the transcription start sites (TSS) of estrogen-induced genes as well as at distal ERα binding sites in an estrogen-dependent manner. Interestingly, H4K12ac occupancy highly correlates with BRD4 binding and enhancer RNA production on ERα-positive enhancers. Consistent with an importance in estrogen-induced gene transcription, H4K12ac occupancy globally increased in ER-positive cells relative to ER-negative cells and these levels were further increased by estrogen treatment in an ERα-dependent manner. Together, these findings reveal a strong correlation between H4K12ac and BRD4 occupancy with estrogen-dependent gene transcription and further suggest that modulators of H4K12ac and BRD4 may serve as new therapeutic targets for hormone-dependent cancers.

## INTRODUCTION

The estrogen receptor-alpha (ERα) plays a central role in determining a luminal epithelial phenotype and tumor progression in a large fraction of breast cancers. Notably, interfering with ERα-regulated gene transcription represents a major therapeutic target in breast cancers where anti-estrogen therapies are the primary indicated therapy for patients with ERα-positive tumors. Estrogen-mediated gene induction is tightly and dynamically regulated. Stimulation with estrogen induces the binding of ERα to estrogen response elements (EREs), which act as enhancers in a manner largely dependent upon the pioneer factor, Forkhead protein A-1 (FOXA1) [1, 2]. Upon activation, ERα further recruits coactivator proteins of the p160 family of histone acetyltransferases (HATs) including SRC1/NCOA1 [3]. SRC1 further interacts with and recruits additional HATs such as p300 and CBP [4, 5]. Additionally, ERα also recruits another HAT, p300/CBP-associated factor (PCAF) [6]. Recruitment of HATs to EREs promotes histone acetylation and thereby leads to gene induction [7]. In particular, acetylation of H3K27 and H4K16 are found near transcriptional start sites (TSS) as well as on active enhancer regions where their occupancy is tightly associated with the recruitment of bromodomain protein-4 (BRD4) [8-12].

BRD4 belongs to the bromo- and extraterminal (BET) domain protein family and serves as a major epigenetic reader of histone acetylation. BRD4 preferentially binds to multiple acetylated lysine residues including K5, K8, K12 and K16 of histone H4 [9, 13] and functions to recruit and activate Cyclin-Dependent Kinase-9 (CDK9), the kinase component of Positive Transcription Elongation Factor-b (P-TEFb) [14]. CDK9 in turn promotes transcriptional elongation by phosphorylating serine 2 of the C-terminal heptapeptide repeat of RNA polymerase (RNAPII) as well as subunits of the Negative Elongation Factor (NELF) and DRB Sensitivity-Inducing Factor (DSIF) complexes [15-17]. Notably, RNAPII Ser2 phosphorylation serves as a hallmark for transcriptional elongation and serves as a platform for the co-transcriptional recruitment of chromatin-modifying enzymes including the RNF20/40 ubiquitin ligase complex, which catalyzes the monoubiquitination of histone H2B at lysine 120 (H2Bub1) in the transcribed region of active genes [16, 18, 19]. Addition of ubiquitin is hypothesized to topologically open chromatin structure [20] and promote transcriptional elongation [16, 19, 21]. In addition to its role in recruiting CDK9 to acetylated chromatin, BRD4 has also been reported to exhibit intrinsic kinase activity and directly phosphorylate RNAPII-PSer2 [22]. These findings support a role for BRD4 in promoting gene expression by binding to acetylated histones and promoting RNAPII elongation in a chromatin context. This effect appears to be, at least in part, dependent upon CDK9 and BRD4 recruitment to enhancers [10, 23] where they promote the transcription of noncoding RNAs from enhancer elements (eRNAs) which are required for induced gene transcription and chromosomal looping [10, 24-27].

A number of studies have uncovered an essential role for BRD4 in various malignancies including Myc-driven cancers [28, 29], leukemia [30, 31], lymphoma [32], lung adenocarcinoma [33], prostate [34] and breast cancers [10, 35, 36]. BRD4 was also reported to regulate metastasis in breast cancer [37]. Consistently, a BRD4-regulated gene signature was reported to predict outcome and survival in breast cancer, especially ER-positive breast cancer [35, 37]. Moreover, BRD4 is required for the growth of ERα-positive tamoxifen-resistant breast cancer where it functions to promote ERα-dependent gene transcription [36]. In addition to these findings, our recent studies also show that BRD4 and downstream histone H2B monoubiquitination are central regulators of estrogen-responsive transcription [10, 38, 39]. BRD4 is recruited to promoters and enhancers of ERα-dependent genes following estrogen stimulation to regulate estrogen-induced transcription and is required for estrogen-dependent proliferation [10]. However, the epigenetic mechanisms controlling BRD4 recruitment to estrogen responsive genes and EREs is poorly understood.

In this study, we examined the association of H4K12ac with BRD4 occupancy genome-wide and analyzed its function in estrogen-regulated transcription. We show that H4K12ac occupies estrogen-responsive gene promoters and EREs in an inducible manner [10] where its occupancy significantly correlates with BRD4 binding, H2Bub1 occupancy, mRNA expression as well as eRNA synthesis. We also observed higher global levels of H4K12ac in ERα-positive breast cancer cells compared to ERα-negative mammary epithelial cells and a further estrogen-dependent increase in ERα-positive cells which was decreased by anti-estrogen treatment. Together these results identify H4K12ac as a potential important epigenetic mediator of ERα activity, possibly via the recruitment of BRD4.

## RESULTS

### H4K12ac correlates with BRD4 binding and active transcription on estrogen-induced genes

**Figure 1:**
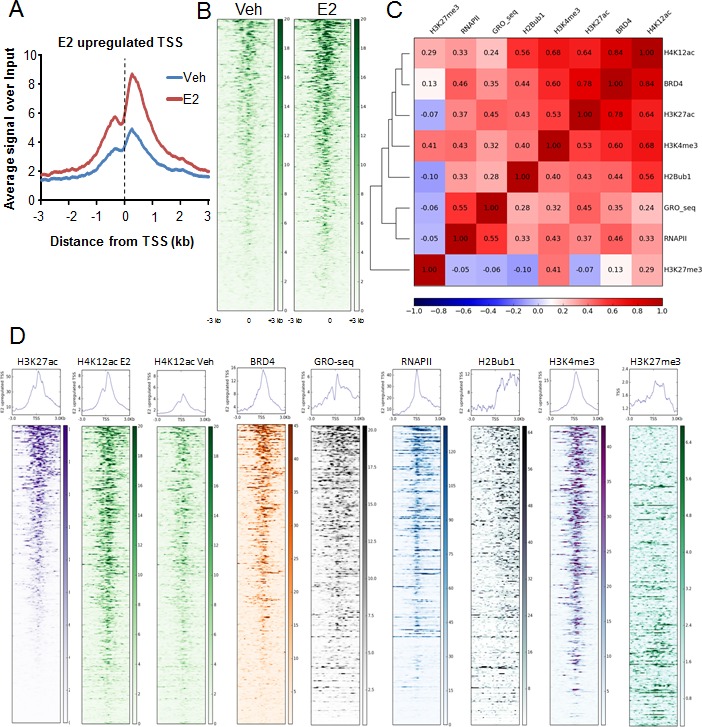
H4K12ac correlates with BRD4 binding in estrogen-induced transcription (A) Average genomic binding profiles of H4K12ac around TSS and 3 kb upstream and downstream of estrogen-induced genes under vehicle (Veh) and estrogen-treated (E2) conditions. The X-axis shows the distance from the TSS of the genes in kilobase pairs. TSS is indicated by a black dotted line. (B) Heatmaps showing genomic binding profiles of H4K12ac around TSS and 3 kb upstream and downstream of estrogen-induced genes under vehicle (Veh) and estrogen-treated (E2) conditions. Center of the heatmap represents TSS. Color key of the heatmaps is shown on the side. (C) Correlation plot showing the heatmap with the Pearson's correlation coefficient values for H4K12ac, BRD4, H3K27ac, H3K4me3, H2Bub1, GRO-seq, RNAPII and H3K27me3 on TSS and 3 kb downstream region of estrogen-upregulated genes. Color key of the heatmap is shown at the bottom of the plot. (D) Heatmaps showing genomic binding profiles of H3K27ac, H4K12ac with estrogen treatment (H4K12ac E2) and without estrogen treatment (H4K12ac Veh), BRD4, nascent RNA transcription (GRO-seq), RNAPII, H2Bub1, H3K4me3 and H3K27me3 around TSS and 3 kb upstream and downstream of estrogen-induced genes. Density of the signals is arranged according to average H3K27ac signals from high to low. Center of the heatmap represents TSS. Color key of the heatmaps is shown at their side.

In order to determine whether H4K12ac may play a role in estrogen-responsive gene expression, we performed genome-wide ChIP-sequencing analyses of H4K12ac with or without estrogen treatment in the ERα-positive MCF7 luminal breast cancer cell line. Aggregate profiles and heatmap analyses showed that estrogen induction increases H4K12ac occupancy near the transcription start sites (TSS) (Fig. 1A-B, Fig. S1A-B). Interestingly, this resembled the increased recruitment of BRD4 adjacent to TSS of estrogen-responsive genes [10]. We further performed correlation and heatmap analyses to determine whether H4K12ac occupancy is correlated with BRD4 [10] recruitment, RNAPII [40] occupancy as well as various other histone modifications. Specifically, we compared H4K12ac occupancy to that of H3K27ac [41] and H3K4me3 [42] which are both active marks of transcription initiation, H2Bub1 [10] which correlates with transcriptional elongation and is dependent upon BRD4 activity [10] and GRO-seq (Global run on-sequencing) [24] which represents nascent RNA transcription. H3K27me3 [42] was used as a negative control and a repressive mark for transcription. Heatmaps were arranged in descending order according to the H3K27ac signals near TSS (TSS and 3 kb downstream). These analyses revealed a high correlation between H4K12ac and BRD4 occupancy and to a lesser extent with H3K27ac occupancy (Fig. 1C-D, Fig. S1C-D). Furthermore, consistent with their common association with active gene transcription, H3K4me3 and H2Bub1 displayed highest correlations with H4K12ac and BRD4. Interestingly, H4K12ac levels did not decrease near the TSS of estrogen-downregulated genes (Fig. S1D-E). These effects are consistent with the negligible change in BRD4 binding on estrogen-repressed genes after estrogen treatment and indicate that histone deacetylase-mediated removal of H4K12ac may occur slower than the transcriptional repression of these genes [10]. Altogether these results uncover a high correlation between H4K12ac and BRD4 occupancy at TSS following estrogen treatment.

### H4K12ac is associated with enhancer function

**Figure 2:**
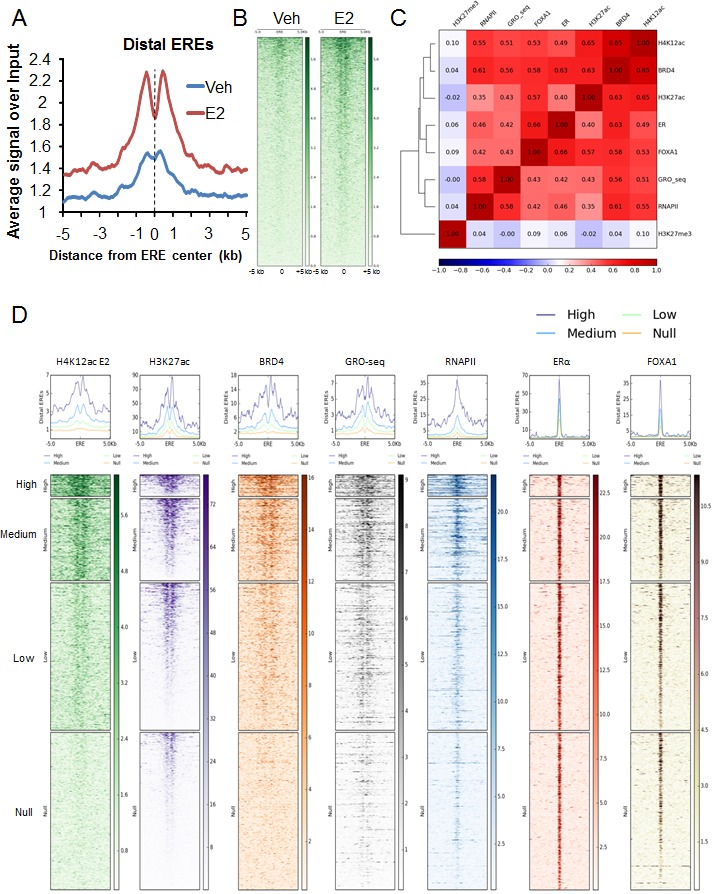
H4K12ac correlates with BRD4 binding in estrogen-induced enhancer function (A) Average genomic binding profiles of H4K12ac around distal EREs and 5 kb upstream and downstream under vehicle (Veh) and estrogen-treated (E2) conditions. X-axis shows the distance from distal EREs in kilobase pairs. Center of ERE is marked with black dotted line. (B) Heatmaps showing genomic binding profiles of H4K12ac around ERE and 5 kb upstream and downstream under vehicle (Veh) and estrogen-treated (E2) conditions. Center of the heatmap represents center of ERE. Color key of the heatmaps is shown on the side. (C) Correlation plot showing the heatmap with the Pearson's correlation coefficient values for H4K12ac, BRD4, H3K27ac, ERα, FOXA1, GRO-seq, RNAPII and H3K27me3 on distal EREs and 1.5 kb upstream and downstream region of estrogen-induced genes. Color key of the heatmap is shown at the bottom of the plot. (D) High, medium, low and null groups were classified according to the H4K12ac signal under estrogen-treated conditions and then heatmaps were plotted for various ChIP-seq signals (H4K12ac E2, BRD4, nascent RNA transcription (GRO-seq), RNAPII, ERα, FOXA1 and H3K27me3) around distal EREs and 5 kb upstream and downstream. Center of the heatmap represents center of the distal ERE region. Color key of the heatmaps is shown on the side.

Given the association of BRD4 with enhancers [23, 32] and its recently established role in promoting eRNA transcription [10, 26, 27], we examined whether H4K12ac occupancy is also preferentially enriched on ERα- and FOXA1-bound enhancers. Indeed, we observed H4K12ac enrichment on distal EREs and this enrichment significantly increased following estrogen treatment as shown in aggregate plot and heatmap analyses (Fig. 2A-B). To further examine the association of H4K12ac at distal ERα-bound enhancers, correlation plot and heatmaps were generated for BRD4, as well as ERα, FOXA1 and H3K27ac on these regions (Fig. 2C, Fig. S2A). We observed that BRD4 and H3K27ac occupancy correlated with H4K12ac on distal enhancers. Notably, apart from its association with FOXA1 and BRD4, ERα binding correlated particularly well with H4K12ac and lesser with H3K27ac. Interestingly, RNAPII occupancy and nascent RNA transcription on enhancers, which are indicative of eRNA production, also correlated with H4K12ac occupancy. Genomic Regions Enrichment of Annotations Tool (GREAT) analyses [43] on distal intergenic regions which exhibited overlapping peaks of H3K27ac, H4K12ac and BRD4 demonstrated an enrichment of estrogen and breast cancer luminal upregulated and basal downregulated-related pathways (Fig. S2B-C). These findings support a strong association among H4K12ac, H3K27ac, BRD4 and ERα function on distal enhancer regions.

To further evaluate the association of H4K12ac with distal enhancer function, distal EREs were grouped into four classes (high, medium, low and null) according to the enrichment of H4K12ac around EREs. Consistent with their potential function as active enhancers, these groups of distal regions also correlated with BRD4 and RNAPII occupancy as well as nascent RNA transcription (Fig. 2D). Together these findings demonstrate that H4K12ac occupies active EREs which are bound by ERα, FOXA1, BRD4 and H3K27ac and produce eRNAs.

### H4K12ac correlates with BRD4 function in gene regulation

**Figure 3:**
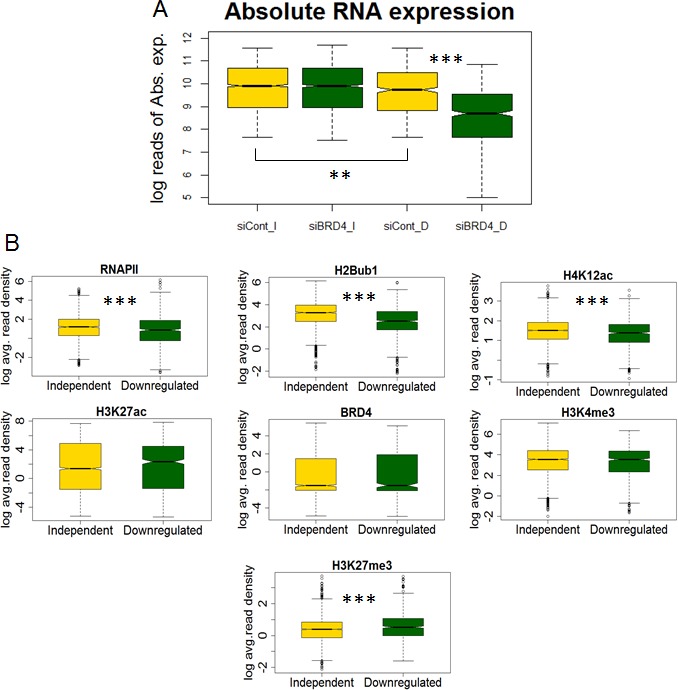
H4K12ac correlates with BRD4 function in regulating gene expression (A) Boxplot showing the absolute gene expression in logarithmic values of normalized counts (log reads of Abs. exp.) under vehicle treated conditions. Genes which are independent (I) and downregulated (D) following BRD4 depletion (siBRD4) are shown. siCont refers to negative control siRNA treated samples. p-values were calculated using Mann-Whitney test and are shown only for significant changes. ***p ≤ 0.001; **p ≤ 0.01; *p ≤ 0.05; (B) Boxplots showing the average ChIP-seq signal in logarithmic values (log avg. read density) of RNAPII, H2Bub1, H4K12ac, H3K27ac, BRD4, H3K4me3 and H3K27me3 under vehicle-treated conditions. Genes which are independent (I) and downregulated (D) following BRD4 depletion (siBRD4) are shown. siCont refers to negative control siRNA treated samples. p-values were calculated using Mann-Whitney test were shown only for significant changes. ***p ≤ 0.001; **p ≤ 0.01; *p ≤ 0.05.

In order to investigate a potential association with the function of BRD4, the average intensity of BRD4, H4K12ac, H3K27ac, H3K4me3, H2Bub1 and RNAPII downstream of the TSS (TSS and 3 kb downstream) were calculated on genes whose expression either decreased (“downregulated”) or was unaffected (“independent”) upon BRD4 depletion under vehicle treated conditions from our previously published RNA sequencing data. Interestingly, genes whose expression decreased following BRD4 depletion were lower expressed than non-regulated genes prior to knockdown (Fig. 3A) and also displayed lower levels of RNAPII (Fig. 3B). Consistent with lower RNAPII occupancy, H4K12ac and H2Bub1 occupancy were also lower on BRD4-dependent genes compared to BRD4-independent genes (Fig. 3B). These results further suggest a cooperative function between H4K12ac and BRD4 in controlling RNAPII and co-transcriptional H2B monoubiquitination to regulate a defined subset of inducible genes.

### H4K12ac occupancy correlates with gene expression levels

**Figure 4:**
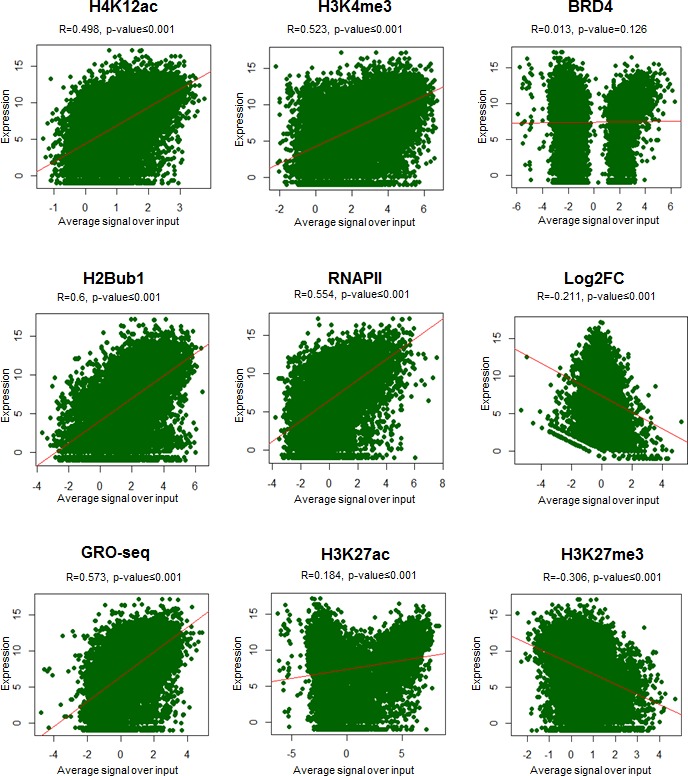
H4K12ac positively correlates with gene expression Scatterplots showing the relationship between various ChIP-seq signals (average signal over input of H4K12ac, H3K4me3, BRD4, H2Bub1, RNAPII, GRO-seq, H3K27ac, H3K27me3, logarithmic fold changes of siBRD4 compared to siCont (Log2FC)) and absolute gene expression in logarithmic values. Pearson correlation coefficient (R) values and two-tailed p-values were shown above the plot. Each dot in the plot represents a single gene. Red line indicates the correlation.

In order to further validate the association of H4K12ac with active gene transcription, average signals of the active transcription marks around TSS were compared with the normalized counts of expression for each gene. As shown in scatterplots along with the respective correlation coefficients, H2Bub1, RNAPII, H3K4me3 and H4K12ac significantly correlated with mRNA expression (Fig. 4, Fig. S3-4). Together these results support a model in which a H4K12ac-BRD4-RNAPII-H2Bub1 axis directs overall gene expression, especially in estrogen-induced transcription.

### Estrogen promotes global H4K12ac

**Figure 5:**
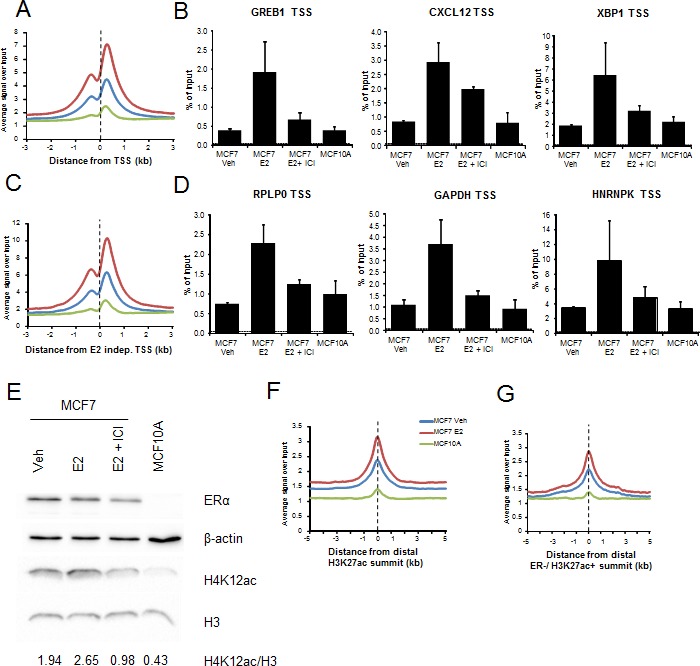
H4K12ac depends upon ERα activity (A, C, F, G) Average genomic binding profiles of H4K12ac in vehicle- (Veh-blue) and estrogen-treated (E2-red) MCF7 or MCF10A (green) cells around TSS and 3 kb upstream and downstream of estrogen upregulated genes (A), TSS and 3 kb upstream and downstream of estrogen-independent genes (C) distal H3K27ac-positive regions and 5 kb upstream and downstream of them (F), and distal H3K27ac-positive regions which don't possess ERα binding (ER negative (−ve)) and 5 kb upstream and downstream of them (G). X-axis shows the distance from the TSS (A, C) or center of the distal H3K27ac-positive region (B, D) in kilobase pairs. TSS or center of H3K27ac peak is indicated by a black dotted line. (B, D) ChIP-qPCR analyses of H4K12ac in vehicle- (Veh), estrogen-treated (E2) and/or ICI-182,780 (E2+ ICI) treated MCF7 or MCF10A cells on TSS of respective estrogen-dependent (GREB1, CXCL12 and XBP1) (B) and estrogen-independent genes (RPLP0, GAPDH and HNRNPK) (D). H4K12ac enrichment is denoted by percentage of input values. Dotted line indicates background DNA precipitated by negative control IgG antibody. (E) Western blot analyses showing ERα, β-actin, H4K12ac and H3 levels in vehicle- (Veh), estrogen-treated (E2) and/or ICI-182,780 (E2+ ICI) treated MCF7 or MCF10A cells. H4K12ac levels were normalized with H3 and the values were shown under the blot.

As H4K12ac occupancy is closely associated with ERα-dependent gene activity, we also compared H4K12ac occupancy in ERα-positive MCF7 breast cancer cells to that in the ER-negative MCF10A normal mammary epithelial cell line both in ChIP and in whole cell protein lysates. Both single gene ChIP-qPCRs as well as genome-wide ChIP-seq analyses displayed induced H4K12ac occupancy in MCF7 comparing to MCF10A around TSS of estrogen-dependent genes (Fig. 5A-B). Surprisingly, this effect was also observed near the TSS of estrogen-independent genes (Fig. 5C-D) as well as at both distal H3K27ac-positive regions from MCF7 and MCF10A and regions occupied by H3K27ac, but not ERα (Fig. 5F-G). These effects required ERα activity, since treatment of MCF7 cells with the anti-estrogen fulvestrant, which rapidly reduces ERα protein levels via proteosomal degradation [44, 45], decreases H4K12ac occupancy on estrogen-dependent and independent genes (Fig. 5B, D). Together, these studies suggested that H4K12ac levels may globally depend upon ERα activity. Consistently, Western blot analyses confirmed that H4K12ac globally increased after estrogen treatment in MCF7 cells and decreased following fulvestrant treatment (Fig. 5E). Notably, H4K12ac levels were the lowest in ER-negative MCF10A cells. These results suggest a potential specific role of H4K12ac in estrogen-induced transcription and correlation with ERα status.

## DISCUSSION

Signal-induced transcription requires a highly coordinated and complex interplay between various transcription factors, post-translational histone modifications, epigenetic readers and chromatin remodelers in order to induce gene expression in a proper temporal and spatial manner. Dysregulation of these mechanisms frequently results in disorders such as cancer. Positive association of H4K12ac with estrogen-induced gene transcription reveals a potentially important and specific role of this modification in promoting ERα-induced gene transcription. According to our model, ERα-directed H4K12 acetylation facilitates the recruitment of BRD4 to both enhancers and promoters. Subsequently, BRD4 recruits CDK9 in order to promote eRNA synthesis at enhancers and transcriptional elongation of ERα-dependent mRNAs. The latter occurs, at least in part via phosphorylation of RNAPII at serine-2 and the subsequent monoubiquitination of histone H2B, which may help to open chromatin structure and facilitate the activity of other chromatin-associated elongation factors [10, 21, 38, 39].

Transcriptional activation is tightly coupled with histone acetylation [46-48]. However, the mechanisms linking histone acetylation and ERα-dependent transcriptional activation are not completely understood. Here, we show that H4K12ac is globally increased across the ERα cistrome in response to estrogen induction. This supports the dynamic nature of histone acetylation including H4K12ac upon estrogen treatment [49-52]. H4K12ac may be catalyzed by p300/CBP [53, 54], which is a critical determinant of ERα-regulated transcription [3, 55]. Thus, our findings not only validate the role of histone acetyltransferases like p300 in hormone-dependent systems [3, 55], but also provide a further potential mechanistic explanation linking H4K12ac to BRD4 recruitment to estrogen-responsive enhancers and TSS. Thus, H4K12ac may play a central role in transcriptional activation which links ERα binding to the subsequent recruitment of other epigenetic regulators like BRD4, which are essential for ERα activity.

The transcriptional mechanisms controlling stimuli-induced gene expression is tightly coupled to the rapid association and disassociation of transcriptional factor and cofactor complexes as well as dynamic changes in chromatin remodeling and histone modifications [7, 56-58]. Interestingly, we show that the genes which are dependent upon BRD4 exhibit reduced expression and lower RNAPII occupancy than BRD4-independent genes. This could support the possibility that these genes may have a higher turnover of the transcriptional activation complexes. Consistent with the lower expression and RNAPII occupancy, H4K12ac and H2Bub1 occupancy on these genes were also lower compared to BRD4-independent genes and their levels were highly dynamic during estrogen-induced transcription. Pathway analyses of BRD4-regulated genes [10] as well as genes associated with enhancer regions which are co-bound by H3K27ac, H4K12ac and BRD4 revealed a specific enrichment of estrogen-induced genes. Consistently, estrogen-responsive genes display a highly dynamic and ERα-dependent assembly and disassembly of transcription factor and co-factor complexes which determine the magnitude and duration of gene induction [56]. Together these results suggest that estrogen-inducible genes may differentially depend upon BRD4 and its associated up- and downstream epigenetic regulatory mechanisms are based on a necessity for dynamic recruitment of transcriptional cofactors, RNAPII, post-translational modification of histones and chromatin remodeling.

Previous studies have suggested that BRD4 predominantly binds to histone H4 acetylated at lysine residues 5, 8, 12 and 16 [9, 13]. However, the close relationship between histone acetylation and BRD4 in regulating transcriptional activity is less established [11]. Our studies demonstrate a strong link between the dynamic nature of H4K12ac and BRD4 and their association with RNAPII and H2Bub1 during target gene activation.

Induced occupancy of H4K12ac at both TSS and enhancers in cancer cells support a role for histone acetylation and enhancer activity in ERα-dependent cancer progression. Moreover, we describe a significant correlation between histone acetylation and eRNA production, which can control targeted gene induction in various disease-specific conditions [10, 24-26]. Notably, given the potential utility of BET domain inhibitors for the treatment of various cancers [23, 28-30, 32, 33], these findings provide additional insight into the molecular mechanisms by which BRD4 functions in ERα-regulated gene transcription. Considering the recent demonstration of the utility of BET inhibition in the treatment of tamoxifen-resistant and hormone-dependent cancers [10, 34, 36], our studies provide a better understanding into the mechanism controlling BRD4 activity in this system. Importantly, BRD4 inhibition along with Fulvestrant sensitizes tumors for their growth inhibition in tamoxifen-resistant xenograft models [36] and our studies show that Fulvestrant rapidly reduces H4K12ac. This opens up a possibility of significant H4K12ac occupancy in tamoxifen-resistant breast cancers bringing up the therapeutic importance of H4K12ac and combined therapies with BRD4 inhibition and antiestrogens in these cancers. Combination of HDAC inhibitors with tamoxifen is implicated in reversing tamoxifen/aromatase inhibitor-resistance in ER-positive breast cancers and HDAC inhibitors were shown to increase the sensitivity of cells to BRD4 inhibition for blocking cell growth and promoting apoptosis in leukemia and lymphoma to [59-61]. This may potentially provide a rationale for testing specific modulators of BRD4 recruitment to chromatin (e.g., inhibitors of histone deacetylases or histone acetyltransferases which particularly modulate H4K12ac) in regulating hormone-dependent gene expression.

## MATERIALS AND METHODS

### Cell culture

MCF7 cells were kindly provided by K. Effenberger (University Medical Center, Hamburg-Eppendorf) and were grown in phenol red-free high-glucose Dulbecco's modified Eagle's media (DMEM, Invitrogen) supplemented with 10% fetal bovine serum (Thermo Scientific), 1% sodium pyruvate, and 1% penicillin/streptomycin (Sigma-Aldrich) at 37°C. For estrogen induction, cells were deprived of hormones by treating them with DMEM media supplemented with 5% charcoal-dextran-treated fetal bovine serum (CSS; Biochrome), 1% sodium pyruvate, and 1% penicillin/streptomycin after 24 hr of growth. After 48 hr, cells were treated with 10 nM 17 β-estradiol (Sigma-Aldrich) and/or 1 μM ICI-182,780 (Fulvestrant) for 2 hr. MCF10A cells were kindly provided by M. Oren (Weizmann Institute of Science, Israel) and were grown in phenol-red free DMEM/F12 medium supplemented with 5% horse serum, 20 ng/mL epidermal growth factor, 0.1 μg/mL Cholera toxin, 10 μg/mL insulin, 0.5 μg/mL hydrocortisone, 1% penicillin/streptomycin at 37°C.

### Protein isolation and western blotting

As mentioned previously [10, 39], whole cell protein lysates were prepared from MCF7 and MCF10A by incubating the cells in Radioimmuno-precipitation buffer (RIPA buffer - 1% (v/v) NP-40, 0.5% sodium deoxycholate and 0.1% SDS in 1X PBS along with protease inhibitors: 1 mM Pefabloc, 1 ng/μL Aprotinin/Leupeptin, 10 mM β-glycerophosphate and 1 mM N-ethylmaleimide) for 10 minutes and scraping the cells if required. Samples were briefly sonicated to release the chromatin associated proteins. Samples were heated along with SDS loading dye by incubating them at 95 °C for 5-10 minutes. These samples were run in a SDS- Polyacrylamide Gel Electrophoresis (PAGE). Proteins were visualized by Western blotting with following antibodies and dilutions: β-actin, I-19, sc1616, HRP-conjugated, 1:5000, Santa Cruz; ERα, HC-20, sc-543, rabbit polyclonal, 1:1,000, Santa Cruz; H4K12ac, ab61238, rabbit polyclonal, 1:1000, abcam; H3, ab10799, mouse monoclonal, 1:5000, abcam. β-actin was used as loading control for ERα and histone H3 for H4K12ac. Quantification of proteins in Western blot was done using Biorad Image Lab 5.2 software and H4K12ac was normalized with H3.

### ChIP-Seq, RNA-seq and raw data information

Chromatin immunoprecipitation of H4K12ac was performed as described previously [10] using anti-acetyl-histone H4 (Lys12) (07-595; EMD Millipore) and negative control IgG (ab37415; abcam) with 10 min crosslinking with 1% formaldehyde. The following primers were used for ChIP-qPCR analyses: GREB1 TSS Forward - 5′-GCCAAATGGAAGAAGGACAG-3′, Reverse - 5′-ACCACCTACCTCCAGTCACC-3′; CXCL12 TSS Forward – 5′-GCAGTGCGCTCCGGCCTTT-3′, Reverse - 5′-CCTCACTGCAGACCGGGCCA-3′; XBP1 TSS Forward – 5′-ATCCCCAGCTCTGGTCATCT-3′, Reverse- 5′-GCCCAGGGCTCTTTTCTGTA-3′; RPLP0 TSS Forward – 5′-CTTCGCGACCCTACTTAAAGG-3′, Reverse – CAATCAGAAACCGCGGATAG-3′; GAPDH TSS Forward – 5′-CGGCTACTAGCGGTTTTACG-3′, Reverse – 5′-AAGAAGATGCGGCTGACTGT-3′; hnRNPK adjacent to TSS Forward- 5′- TCCACGAGGTCCCTAGTTCC-3′, reverse – 5′- GCCATTTCCCTGAGCGTGTA-3′.

ChIP-seq libraries were made using the NEBNext Ultra DNA library preparation kit according to the manufacturer's instructions [10]. The size range of the libraries was verified to be 250-600 bp using Bioanalyzer 2100. 75 bp single-ended tags were sequenced with single indexing using Illumina NextSeq 500 (XCelris Genomics, Ahmedabad, India). Previously published data for BRD4, H2Bub1 and ERα ChIP-seq as well as RNA-seq are available from the NCBI Gene Expression Omnibus (GEO) (GSE55921, GSE55922) [10]. Raw data for FOXA1, H3K4me3, H3K27me3 [42], H3K27ac [41], RNAPII [40], and GRO-seq [24] were downloaded from the European Nucleotide Archive. Normalization of RNA-sequencing read counts for each gene was performed using DESeq [62].

### Bioinformatic analyses

ChIP-seq reads were mapped to the human reference genome (UCSC HG19) using Bowtie (version 1.0.0) [63]. Sam files from Bowtie were converted into bam files by SAMtools [64]. DeepTools [65] was used to create bigwigs by normalizing the ChIP-seq sample with the input samples of each cell line and obtaining the ratio of the reads using bamCompare function. Normalization was done using read counts of each ChIP-seq sample. Average density was calculated using computeMatrix function in deepTools surrounding TSS (plus 3 kb) or ERE (±1.5 kb). The Heatmapper function from deepTools was used to create heatmaps and average profiles. TSS and gene body coordinates for hg19 were obtained from UCSC Table Browser [66]. Estrogen-upregulated, downregulated and BRD4 siRNA downregulated and BRD4-independent genes were categorized from our previously published RNA seq datasets [10]. Distal EREs or H3K27ac regions were obtained from ERα or H3K27ac binding sites which were not within gene bodies and regions 5 kb upstream or downstream of them. TSS and 3 kb downstream regions were used for TSS-based correlation plots and 1.5 kb up- and downstream of distal EREs for distal ERE-based correlation plots. Correlation plots were made using the bamCorrelate function in deepTools. Groups based on H4K12ac signals were determined using k-means clustering in heatmapper function of deepTools. Molecular signature database (MSigDB) [67] pathways whose target genes are enriched for BRD4 or specific histone modifications were identified using the Genomic Regions Enrichment of Annotations Tool (GREAT) [43]. Boxplots and scatterplots were made using R for statistical computing. The Mann-Whitney test was used to calculate the statistical significance for boxplots. Pearson correlation coefficient values were calculated using R for scatter plots and p-values were calculated using two-tailed probability test.

## SUPPLEMENTARY MATERIAL, FIGURES


