# Supplementary material for: H4K12ac is regulated by estrogen receptor-alpha and is associated with BRD4 function and inducible transcription
Source: Oncotarget. 2015 Jan 31;6(9):7305–17. doi: 10.18632/oncotarget.3439 (PMC4466686; doi:10.18632/oncotarget.3439)
Supplement: Supplementary file 1 [file oncotarget-06-7305-s001.pdf]

H4K12ac is regulated by estrogen receptor-alpha and is associated with BRD4 function and inducible transcription

Supplementary Material

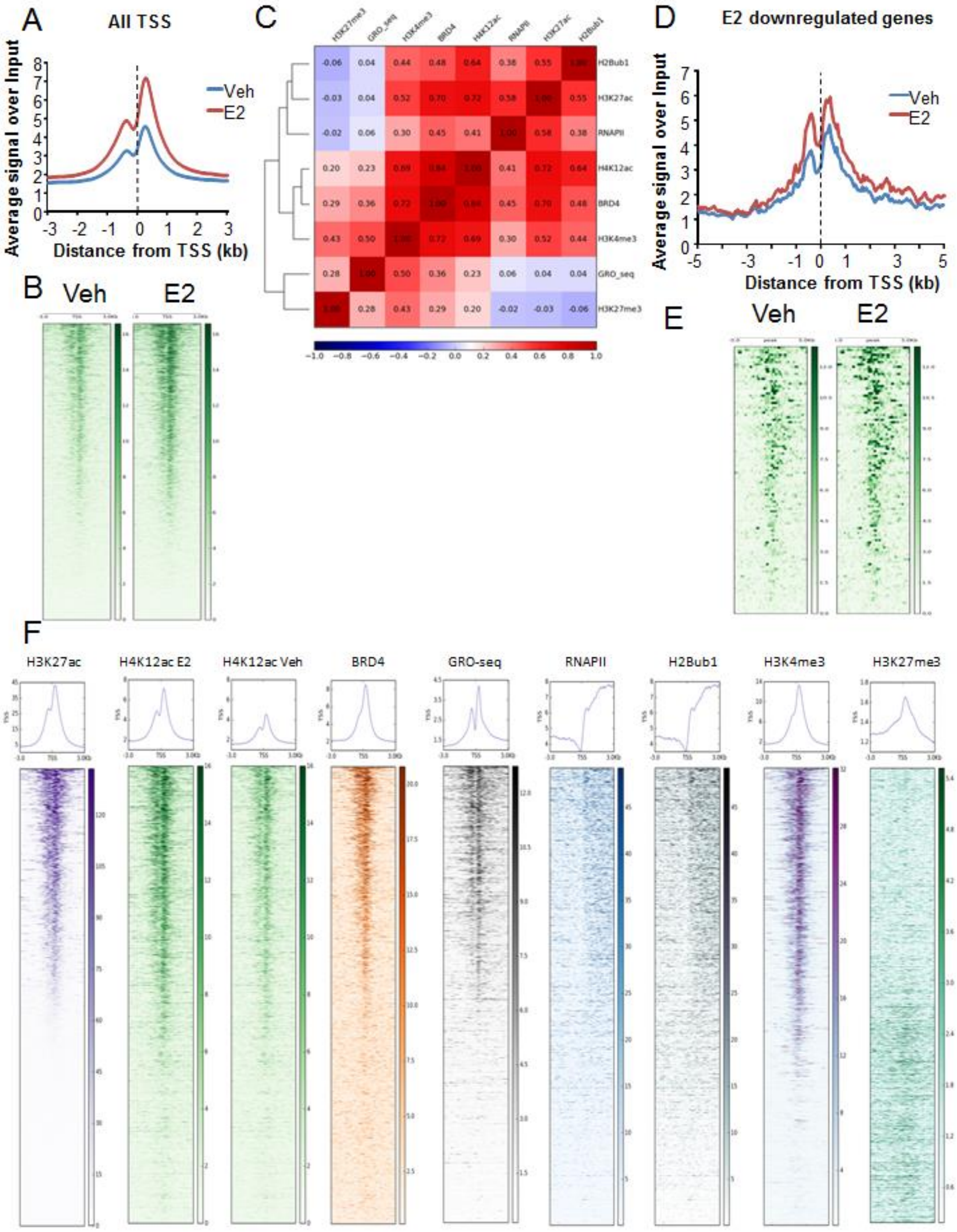

Figure S1: (A) Average genomic binding profiles of H4K12ac around TSS and 3 kb upstream and downstream of all the genes under vehicle (Veh) and estrogen-treated (E2) conditions. The X-axis shows the distance from the TSS of the genes in kilobase pairs. TSS is indicated by a black dotted line. (B) Heatmaps showing genomic binding profiles of H4K12ac around TSS and 3 kb upstream and downstream of all genes under vehicle (Veh) and estrogen-treated (E2) conditions. Center of the heatmap represents TSS. Color key of the heatmaps is shown on the side. (C) Correlation plot showing the heatmap with the Pearson's correlation coefficient values for H4K12ac, BRD4, H3K27ac, H3K4me3, H2Bub1, GRO-seq, RNAPII and H3K27me3 on TSS and 3 kb downstream region of all genes. Color key of the heatmap is shown at the bottom of the plot. (D) Average genomic binding profiles of H4K12ac around TSS and 3 kb upstream and downstream of estrogen-downregulated genes under vehicle (Veh) and estrogen-treated (E2) conditions. The X-axis shows the distance from the TSS of the genes in kilobase pairs. TSS is indicated by a black dotted line. (E) Heatmaps showing genomic binding profiles of H4K12ac around TSS and 3 kb upstream and downstream of all genes under vehicle (Veh) and estrogen-treated (E2) conditions. Center of the heatmap represents TSS. Color key of the heatmaps is shown on the side. (F) Heatmaps showing genomic binding profiles of H3K27ac, H4K12ac with estrogen treatment (H4K12ac E2) and without estrogen treatment (H4K12ac Veh), BRD4, nascent RNA transcription (GRO-seq), RNAPII, H2Bub1, H3K4me3 and H3K27me3 around TSS and 3 kb upstream and downstream of estrogen-induced genes. Density of the signals is arranged according to average H3K27ac signals from high to low. Center of the heatmap represents TSS. Color key of the heatmaps is shown at their side.

A

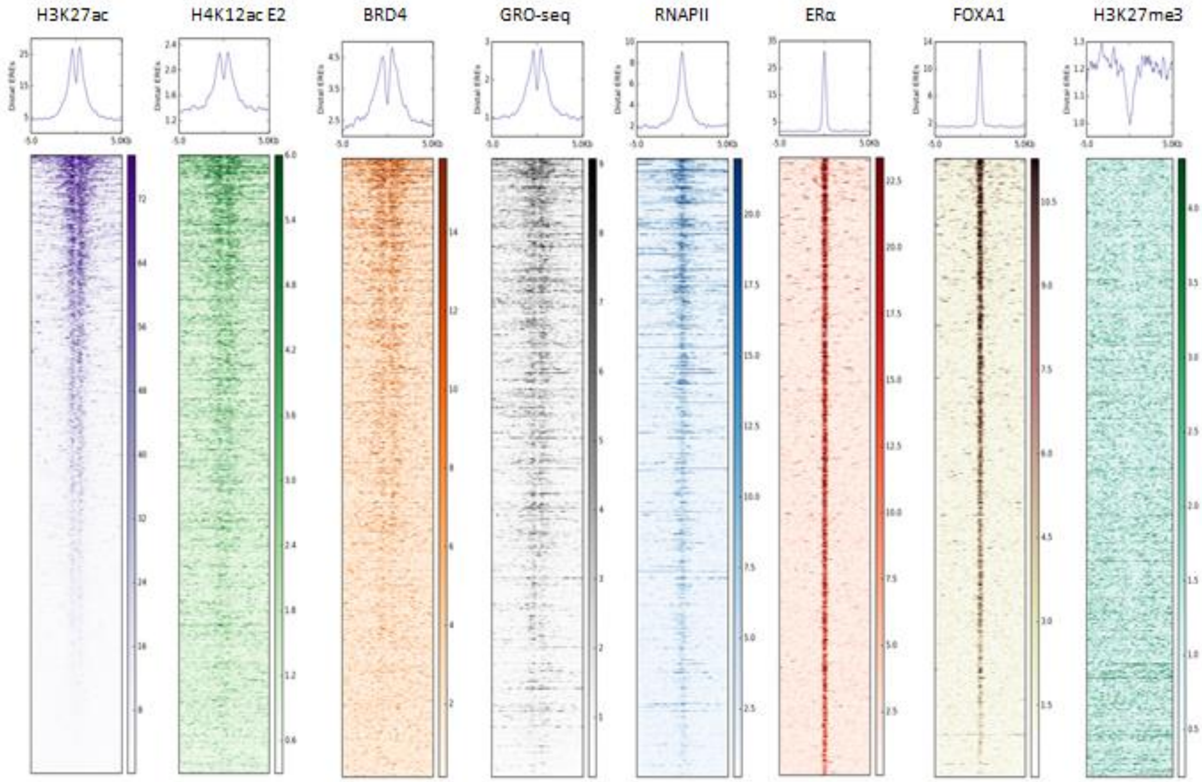

B

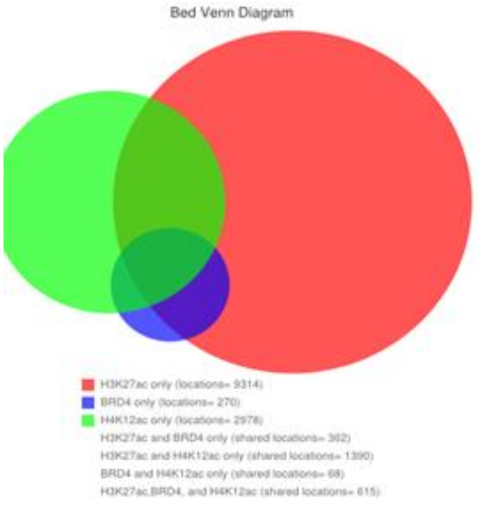

C

MSigDB ID	-log10(Binomial p value)
GARY_CD5_TARGETS_UP	13.52352575
HALMOS_CEBPA_TARGETS_DN	13.33967441
ENK_UV_RESPONSE_KERATINOCYTE_UP	12.62124881
MITSIADES_RESPONSE_TO_APLIDIN_UP	12.48514476
UDAYAKUMAR_MED1_TARGETS_DN	11.28121698
SPIELMAN_LYMPHOBLAST_EUROPEAN_VS_ASIAN_UP	10.86018449
LOPEZ_MESOTHELIOMA_SURVIVAL_OVERALL_DN	10.66214935
LUI_THYROID_CANCER_CLUSTER_1	10.57032474
LEONARD_HYPOXIA	10.49309238
AMIT_EGF_RESPONSE_40_HELA	10.48841179
MANALO_HYPOXIA_UP	10.20579563
SMID_BREAST_CANCER_RELAPSE_IN_LIVER_DN	9.951153225
BENPORATH_MYC_TARGETS_WITH_EBOX	9.541881349
KIM_WT1_TARGETS_DN	9.410117725
STEIN_ESR1_TARGETS	9.347385294
NUYTTEN_NIPP1_TARGETS_DN	9.187657414
CHARAFE_BREAST_CANCER_LUMINAL_VS_MESENCHYMAL_UP	9.173701107
LIU_CDX2_TARGETS_DN	9.132821234
STEIN_ESTROGEN_RESPONSE_NOT_VIA_ESRRA	9.099766622
MILI_PSEUDOPODIA_HAPTOTAXIS_DN	9.000357398
MOHANKUMAR_TLX1_TARGETS_UP	8.956431432
AMIT_DELAYED_EARLY_GENES	8.784263588
BLUM_RESPONSE_TO_SALIRASIB_UP	8.718630696
SMID_BREAST_CANCER_BASAL_DN	8.598820282
WANG_CISPLATIN_RESPONSE_AND_XPC_UP	8.350592797
DIRMEIER_LMP1_RESPONSE_EARLY	8.195201105
CREIGHTON_ENDOCRINE_THERAPY_RESISTANCE_1	8.186093246
GOZGIT_ESR1_TARGETS_DN	8.132177326
DOANE_BREAST_CANCER_ESR1_UP	7.887568282
VICENT_METASTASIS_DN	7.821348176
WINTER_HYPOXIA_UP	7.709285749
TONKS_TARGETS_OF_RUNX1_RUNX1T1_FUSION_HSC_DN	7.706635456
ELVIDGE_HYPOXIA_UP	7.674113621
FRASOR_RESPONSE_TO_ESTRADIOL_UP	7.645330415

Figure S2: (A) Heatmaps showing genomic binding profiles of H3K27ac, H4K12ac with estrogen treatment (H4K12ac E2) and without estrogen treatment (H4K12ac Veh), BRD4, nascent RNA transcription (GRO-seq), RNAPII, ERα, FOXA1 and H3K27me3 around distal EREs and 5 kb upstream and downstream with all estrogen-treated (E2) conditions. Density of the signals is arranged according to H3K27ac signals from high to low. Center of the heatmap represents center of the distal ERE region. Color key of the heatmaps is shown on the side. (B) Venn diagram showing the overlap of distal binding regions of H3K27ac (red), H4K12ac (green) and BRD4 (blue). (C) GREAT analyses on distal regions which oveelpas with H3K27ac, H4K12ac and BRD4 occupancy. The names of the Molecular signature database (MSigDB) pathways are denoted with their –log 10 binomial p-values. Estrogen-specific pathways are highlighted in pink and other breast cancer-related pathways in brown.

E2 upregulated genes

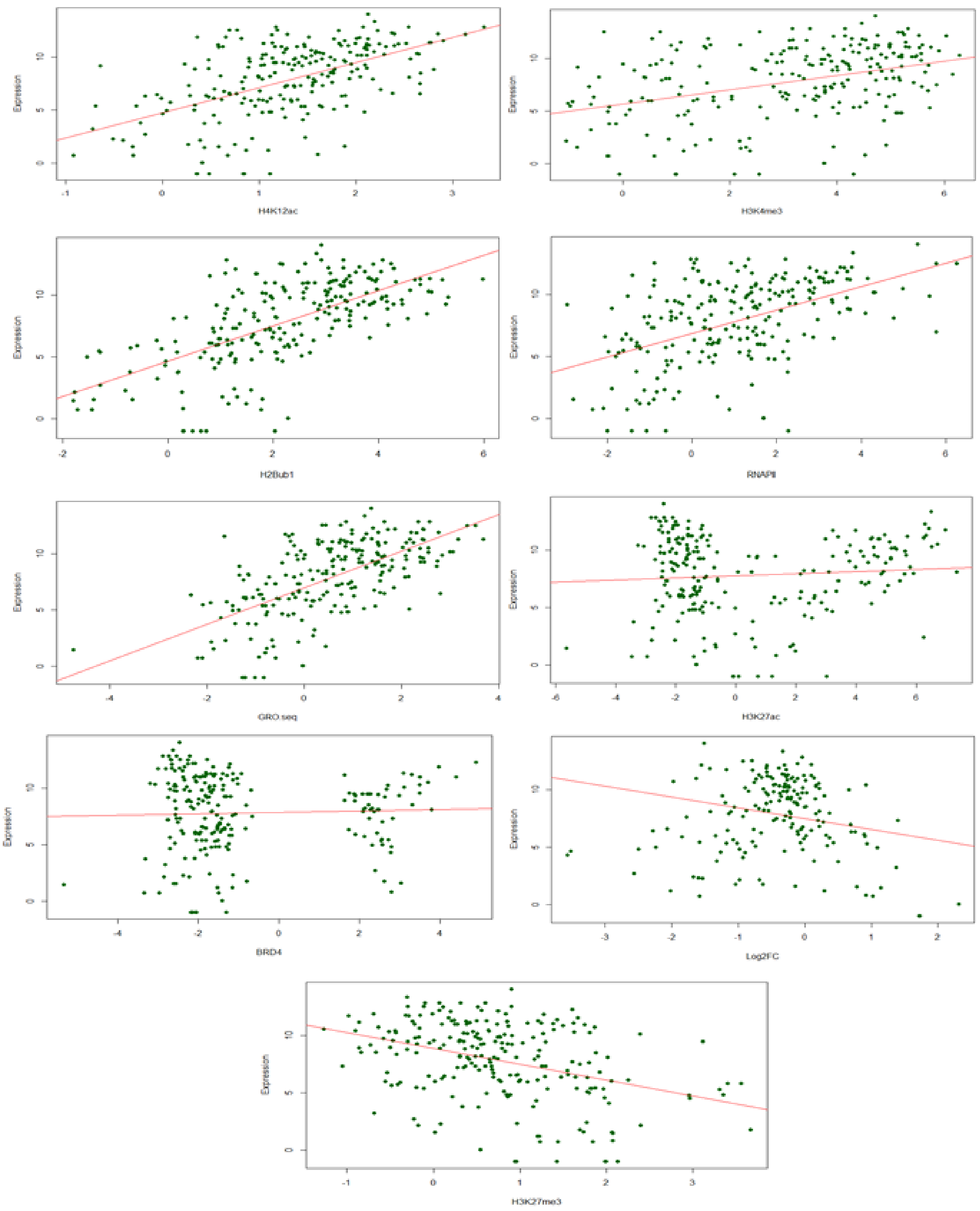

Figure S3: Scatterplots showing the relationship between various ChIP-seq signals (H4K12ac, H3K4me3, BRD4, H2Bub1, RNAPII, GRO-seq, H3K27ac, H3K27me3, logarithmic fold changes of siBRD4 compared to siCont (Log2FC)) and absolute gene expression in logarithmic values for estrogen upregulated genes. Each dot in the plot represents a single gene. Red line indicates the correlation.

All genes

	GRO.seq	H2Bub1	H3K4me3	RNAPII	H3K27me3	BRD4	H3K27ac	H4K12ac	Expression	Log2FC
GRO.seq	1	0.737716	0.607588	0.740766	-0.16243	0.109476	0.29921	0.59734	0.573008	-0.07581
H2Bub1	0.737716	1	0.604958	0.617574	-0.02786	0.093484	0.262579	0.69864	0.600286	-0.08857
H3K4me3	0.607588	0.604958	1	0.623692	0.181553	0.085577	0.288095	0.749786	0.521682	-0.12752
RNAPII	0.740766	0.617574	0.623692	1	-0.165	0.018243	0.206677	0.635878	0.553889	-0.09979
H3K27me3	-0.16243	-0.02786	0.181553	-0.165	1	0.074421	-0.01146	0.069792	-0.30611	0.010353
BRD4	0.109476	0.093484	0.085577	0.018243	0.074421	1	0.675574	0.036245	0.011641	-0.01329
H3K27ac	0.29921	0.262579	0.288095	0.206677	-0.01146	0.675574	1	0.224662	0.183565	-0.04418
H4K12ac	0.59734	0.69864	0.749786	0.635878	0.069792	0.036245	0.224662	1	0.497749	-0.09188
Expression	0.573008	0.600286	0.521682	0.553889	-0.30611	0.011641	0.183565	0.497749	1	-0.21114
Log2FC	-0.07581	-0.08857	-0.12752	-0.09979	0.010353	-0.01329	-0.04418	-0.09188	-0.21114	1

E2 regulated genes

	GRO.seq	H2Bub1	H3K4me3	RNAPII	H3K27me3	BRD4	H3K27ac	H4K12ac	Expression	Log2FC
GRO.seq	1	0.677545	0.536808	0.703225	-0.22096	0.040218	0.112583	0.510785	0.629388	-0.09888
H2Bub1	0.677545	1	0.555719	0.496261	-0.05379	0.136138	0.189556	0.686092	0.640592	-0.02342
H3K4me3	0.536808	0.555719	1	0.572254	0.242915	0.018732	0.233546	0.721774	0.375121	0.029958
RNAPII	0.703225	0.496261	0.572254	1	-0.19608	-0.0191	0.091506	0.540041	0.490185	-0.0855
H3K27me3	-0.22096	-0.05379	0.242915	-0.19608	1	0.050236	0.040627	0.068655	-0.36606	0.081474
BRD4	0.040218	0.136138	0.018732	-0.0191	0.050236	1	0.591603	0.008577	0.038346	-0.21605
H3K27ac	0.112583	0.189556	0.233546	0.091506	0.040627	0.591603	1	0.161019	0.07871	-0.04206
H4K12ac	0.510785	0.686092	0.721774	0.540041	0.068655	0.008577	0.161019	1	0.540699	-0.10892
Expression	0.629388	0.640592	0.375121	0.490185	-0.36606	0.038346	0.07871	0.540699	1	-0.24406
Log2FC	-0.09888	-0.02342	0.029958	-0.0855	0.081474	-0.21605	-0.04206	-0.10892	-0.24406	1

Figure S4: Pearson correlation coefficient (R) values were shown in tables representing the relationship between various ChIP-seq signals (H4K12ac, H3K4me3, BRD4, H2Bub1, RNAPII, GRO-seq, H3K27ac, H3K27me3, logarithmic fold changes of siBRD4 compared to siCont (Log2FC)) and absolute gene expression for all genes and estrogen-induced genes.
